# Evidence for the involvement of the anthranilate degradation pathway in *Pseudomonas aeruginosa* biofilm formation

**DOI:** 10.1002/mbo3.33

**Published:** 2012-09-01

**Authors:** Patricia Costaglioli, Christophe Barthe, Stephane Claverol, Volker S Brözel, Michel Perrot, Marc Crouzet, Marc Bonneu, Bertrand Garbay, Sebastien Vilain

**Affiliations:** 1 Biotechnologie des Protéines Recombinantes à Visée Santé, University BordeauxEA4135, F-33000, Bordeaux, France; 2 Centre Génomique Fonctionnelle de Bordeaux, University BordeauxPôle Protéomique, F-33000, Bordeaux, France; 3 Department of Biology and Microbiology, South Dakota State UniversityBrookings, South Dakota, 57007, USA

**Keywords:** Anthranilate degradation, biofilm, glass wool, *Pseudomonas aeruginosa*

## Abstract

Bacterial biofilms are complex cell communities found attached to surfaces and surrounded by an extracellular matrix composed of exopolysaccharides, DNA, and proteins. We investigated the whole-genome expression profile of *Pseudomonas aeruginosa* sessile cells (SCs) present in biofilms developed on a glass wool substratum. The transcriptome and proteome of SCs were compared with those of planktonic cell cultures. Principal component analysis revealed a biofilm-specific gene expression profile. Our study highlighted the overexpression of genes controlling the anthranilate degradation pathway in the SCs grown on glass wool for 24 h. In this condition, the metabolic pathway that uses anthranilate for Pseudomonas quinolone signal production was not activated, which suggested that anthranilate was primarily being consumed for energy metabolism. Transposon mutants defective for anthranilate degradation were analyzed in a simple assay of biofilm formation. The phenotypic analyses confirmed that *P. aeruginosa* biofilm formation partially depended on the activity of the anthranilate degradation pathway. This work points to a new feature concerning anthranilate metabolism in *P. aeruginosa* SCs.

## Introduction

In natural environments, microorganisms are capable of attaching to different surfaces before forming complex cellular communities known as biofilms (Costerton et al. 1999; Lindsay and Holy 2006). Biofilms are made of aggregated cells, which are embedded in a hydrated matrix composed of extracellular polymeric substances produced by the microorganisms themselves (Hall-Stoodley and Stoodley 2009). Biofilms are often a source of troubles in natural, industrial, and medical settings (Guerrero et al. 2009). In the field of health, they may be the cause of nosocomial diseases (Otto 2008), catheter infections (Jacobsen et al. 2008), or colonization of artificial organs (Litzler et al. 2007). In cystic fibrosis (CF) patients and individuals with compromised immune systems, the colonization and formation of biofilms by *Pseudomonas aeruginosa*, an opportunistic human pathogen, can cause life-threatening infections (Wagner and Iglewski 2008). One characteristic of sessile cells (SCs), which are present in biofilms, is their high level of resistance to environmental stresses and particularly to antibiotic treatments (Hoyle and Costerton 1991; Høiby et al. 2010). The origin of the overall resistance is still a matter of debate, although biofilm-specific gene expression in SCs has been proposed (Mah and O'Toole 2001). These specific genes and corresponding proteins are considered as potential molecular targets to fight against the formation and/or maintenance of biofilms (Landini et al. 2010). Interestingly, antibiotic resistance may occur either in all SCs, or only in a subpopulation of cells called “persisters” (Lewis 2005; De Groote et al. 2009). Thus, a thorough understanding of the biofilm physiology is necessary for the rational design of inhibitors that could prevent the numerous clinical and environmental complications associated with biofilms. Several studies have been devoted to identifying the transcripts or proteins specific to SCs. These studies provided information concerning the mechanisms involved in the colonization of a surface by bacteria, and in the subsequent development and dispersion of biofilms (Kjelleberg and Givskov 2007). Indeed, several transcriptomic studies using DNA microarray technology have investigated different aspects of biofilm physiology (An and Parsek 2007). The large amount of data generated by these studies revealed interesting insights into the biofilm lifestyle. However, despite some overlap among the lists of differentially expressed genes thought to be involved in biofilm formation, the use of different culturing systems has prevented a consensus biofilm transcriptomic signature from emerging, and the key genes involved in the biofilm lifestyle have yet to be identified (An and Parsek 2007). Biofilm-specific proteomes have been reported for the SCs of *P. aeruginosa* (Vilain et al. 2004a) and *Bacillus cereus* (Vilain and Brözel 2006). However, multiple biofilm-specific proteomes exist, which vary according to the nature of the colonized surface (Vilain et al. 2004b). The influences exerted by different colonized surfaces on gene expression could explain the different sets of results obtained by comprehensive studies of the same bacterial species (Beloin and Ghigo 2005). Recently, it was proposed that biofilms are part of a continuum of growth mode, each adapted to specific environmental domains. So, along with the successive growth steps, biofilms may proceed through different, but converging paths (Patell et al. 2010). Thus, it is important to identify the different physiological pathways participating in the formation of bacterial biofilms.

In this work, we investigated the whole-genome expression profile of *P. aeruginosa* SCs in order to identify specific pathways involved in biofilm formation. The experimental system to grow biofilms was based on glass wool colonization (Steyn et al. 2001). This substratum for cell attachment offers a large surface-to-volume ratio. In addition, glass wool permits to bind DNA molecules on its surface (Vilain et al. 2009), which is a component of the extracellular matrix. Here, we have used transcriptomic analyses to characterize the gene expression profiles of sessile *P. aeruginosa* PAO1 cells and compared these profiles to those of planktonic cells. Among the differentially expressed genes, we observed the induction of a specific catabolic pathway that degrades anthranilate to yield succinyl-CoA and acetyl-CoA. This gene overexpression was confirmed at the proteomic level by using isobaric tags for relative and absolute quantification (iTRAQ) technique. To our knowledge, this is the first evidence that increased anthranilate degradation is involved in biofilm formation. To support this view, we demonstrated that *P. aeruginosa* strains harboring mutations in genes encoding enzymes involved in this pathway were impaired in their ability to form biofilms.

## Materials and Methods

### Bacteria and growth conditions

*Pseudomonas aeruginosa* PAO1 (CIP 104116) was provided by the Institut Pasteur (CRBIP, Paris, France). Transposon mutants were obtained from a *P. aeruginosa* transposon mutant library available at the University of Washington Genome Center (Jacobs et al. 2003). All strains were cultivated in lysogeny broth (LB) (tryptone 10 g/L; yeast extract 5 g/L; NaCl 5 g/L, pH 7.2), except the transposon mutants cultivated in LB supplemented with 60 *μ*g/mL tetracycline. Overnight precultures were obtained by inoculating one colony into 3 mL of LB in a 15-mL Falcon tube and growing the culture at 37°C under agitation (150 rpm, Unitron, Infors, Massy, France). Planktonic cell (PC) cultures were started by inoculating 200 mL of LB in a 500 mL Erlenmeyer flask with 10^6^ UFC/mL from a preculture. Cultures were incubated at 37°C under agitation for 4 h (mid-exponential growth phase, 0.3 OD_546nm_) or for 24 h (late stationary growth phase, 5 OD_546nm_). These cultures were termed PC4 and PC24, respectively. Biofilms were cultured in a model system initially described by Steyn and coauthors (2001). Sessile cells were obtained using the same growth conditions described above for PC cultures, except that a 4-g piece of glass wool (Ref. A1908B, Bioblock) sterilized in distilled water, was introduced into the Erlenmeyer flask. Bacterial growth on glass wool lasted for 24 h, and the corresponding culture was termed SC24. Bacterial cultures PC4, PC24, and SC24 were each performed three times, using an independent clone each time.

### Harvest of PC and SCs

Planktonic cells were harvested by centrifugation (8000 × *g*, 10 min, 4°C) and the pellets were washed twice with 0.1 mol/L sodium phosphate buffer (PB) pH 7.2. Sessile cells were harvested as follows: (i) glass wool pieces were removed from the flasks and gently washed twice in PB to eliminate any loosely bound PCs; (ii) SCs (sample A) were harvested from glass wool fibers by five cycles of manual wringing out and absorption in 100 mL of PB (“sponge effect”); (iii) the glass wool was then placed into 50 mL of PB and sonicated (Elma Transsonic sonicator, 35 W) for 30 sec to release adherent SCs (sample B); (iv) finally, the samples A and B were pooled and centrifuged as described for PC cultures. Bacterial pellets were either used immediately for RNA purification or were frozen (−80°C) for protein extraction. Three biological replicates for the cultures of PAO1: PC4 (a, b, c), PC24 (a, b, c), and SC24 (a, b, c) were obtained.

### RNA extraction, microarray analysis, and data processing

Bacterial pellets were immediately resuspended in a mix of PB/RNAprotect Bacteria Reagent (QIAGEN, Hilden, Germany) (1v/2v), and incubated 5 min at room temperature. Treated cells were then centrifuged (10,000 × *g*, 10 min, 4°C), and total RNA was isolated from *P. aeruginosa* using the QIAGEN RNAeasy protocol including on-column DNA digestion. RNA purity and concentration were determined using a NanoDrop ND-1000 spectrophotometer (NanoDrop Technologies, Wilmington, Delaware) and RNA integrity was evaluated using the RNA 6000 NanoChip assay on a 2100 Bioanalyzer (Agilent Technologies, Massy, France).

For the GeneChip experiments, cDNAs were synthesized, fragmented, labeled, and processed as recommended by Affymetrix (Affymetrix, Santa Clara, CA). Hybridizations on GeneChip® *P. aeruginosa* Genome Array and scanning were performed using DNAVision (DNAVision SA, Charleroi, Belgium). Raw data were obtained using the Affymetrix GeneChip operating system 1.4 software, and normalized using the robust multiarray average method. In order to focus on the most significant differences, we determined a threshold value that corresponded to the mean of the six medians of data series (PC24 [a–c] and SC24 [a–c]). Hence, when the signal of the probe ID after normalization was below this threshold (17.5 arbitrary unit) in all samples, the corresponding gene was excluded from the analysis. A gene was classified as “overexpressed” or “underexpressed” if the average of the values measured in the three replicates for SC24 was at least twofold higher or twofold lower, respectively, than the averages measured for PC24. The microarray data have been deposited in the NCBI Gene Expression Omnibus repository under accession number GSE30021.

### Protein preparation and iTRAQ experiments

After thaw, bacterial pellets were suspended in a protein solubilization solution composed of 7 mol/L urea, 2 mol/L thiourea, 50 mmol/L DTT, and 2% (w/v) CHAPS at a ratio of 0.1 g of cells per mL of solution. Cells were lysed with two freeze–thaw cycles (−80 to 25°C) followed by sonication (1 min at 15 W, Branson Sonifier 150). Proteins were obtained by overnight precipitation in 15% TCA (final concentration), followed by two glacial acetone washes. Protein pellets were resuspended overnight at room temperature in 250 *μ*L of a solution of 7 mol/L urea, 2 mol/L thiourea, and 4% (w/v) CHAPS. The protein samples were sonicated for 15 sec twice on ice, and clarified by another 15-min centrifugation at 17,000 × *g*. Supernatants were collected and their protein concentrations were determined using a protein assay according to the manufacturer's instructions (Bio-Rad, France).

For each biological sample, 100 *μ*g of protein diluted in dissolution buffer (500 mmol/L triethylammonium bicarbonate, 4 mol/L urea) was digested with trypsin and labeled with iTRAQ 4-plex as described in the reference guide provided by the supplier (Applied Biosystems, Foster City, California). Labeled samples were pooled and concentrated to a volume of 30 *μ*L in a vacuum centrifuge. This iTRAQ experiment was performed in triplicate (R1, R2, R3), with each replicate containing one biological sample from the PC24 and SC24 cultures. For example, the R1 mixture contained samples from the PC24a and SC24a cultures; R2 contained the b-samples and R3 contained the c-samples. Impurities were removed from the samples using a cation exchange cartridge (Applied Biosystems) before the samples were desalted on a Sep-Pak C18 Light Cartridge (Waters, Milford, Massachusetts). Eluates were concentrated down to a volume of 10 *μ*L in a vacuum centrifuge and subsequently diluted into 380 *μ*L of 8 mol/L urea in the presence of 4.4 *μ*L of IPG, pH 3.5–4.5. Peptide samples were loaded onto an 18-cm IPG strip (GE Healthcare, Uppsala, Sweden), pH 3.5–4.5. The strips were passively rehydrated for 10 h at 20°C in a Protean IEF Cell (Bio-Rad). Separation was performed by applying 500 V for 1 h, followed by 1000 V for 1 h, and 8000 V for a total of 60 kVh. After migration, strips were cut in 32 equal slices and each slice was placed in a well of a polypropylene 96-well plate. Peptides were extracted from the slices by three successive incubations with (i) 0.1% TFA in water, (ii) 50% ACN + 0.1% TFA, and (iii) 100% ACN + 0.1% TFA. For each incubation step, samples were gently mixed for 10 min before sonicating for 2 min in an ultrasonic bath. All three supernatants were pooled and concentrated to a volume of 100 *μ*L in a vacuum centrifuge. Each fraction was diluted by adding 150 *μ*L of 0.1% TFA in water and loaded on Sep-Pak tC18 MicroElution Plate (Waters). Desalted peptides were eluted in 250 *μ*L 90% ACN + 0.1% TFA. Eluates were again concentrated to a volume of 40 *μ*L in a vacuum centrifuge. Peptides were then analyzed by LC-MS/MS.

### NanoLC-MS/MS analysis

Peptide mixtures were analyzed on an Ultimate 3000 Nano LC system (Dionex, Voisins le bretonneux, France) coupled to a nanospray LTQ XL mass spectrometer (ThermoFinnigan, San Jose, California). Ten microliters of peptide digests were desalted onto a 300-*μ*m i.d. × 5-mm C18 PepMap™ trap column (LC Packings Netherlands, Amsterdam, Netherlands) at a flow rate of 30 *μ*L/min and separated onto an analytical 75-*μ*m i.d. × 15-cm C18 PepMap column (LC Packings). Mobile phases were a mix of solvent A (0.1% formic acid in 5% ACN) and solvent B (0.1% formic acid in 80% ACN). Elution was performed using a 5–40% linear gradient of solvent B for 35 min. The separation flow rate was set at 200 nL/min. Data were acquired in a data-dependent mode that alternated between an MS scan survey over an m/z range of 300–1700, and three MS/MS scans with pulsed Q collision-induced dissociation (PQD) as the activation mode. MS/MS spectra were acquired using a 2-m/z unit ion isolation window and a normalized collision energy of 29. MS/MS spectra resulted from three MS/MS microscans. Monocharged ions were rejected and the dynamic exclusion duration was set to 20 sec.

### Database search and processing of results

Data were searched using SEQUEST through a Bioworks 3.1.1 interface (ThermoFinnigan) against the *P. aeruginosa* PAO1 database (version 2008-01-10; 5568 entries), which included sequences in the reverse sense to obtain false-positive rate evaluations. DTA files were generated from MS/MS spectra that reached a minimal intensity (1000) and at least 10 ions. The DTA generation authorized the averaging of several MS/MS spectra corresponding to the same precursor ion with a tolerance of 1.4 amu. Spectra from peptides with molecular masses between 600 and 3500 Da were retained. The search parameters were as follows: mass accuracy of the monoisotopic peptide precursor was set to 2 amu, and that for the peptide fragments was set to 1 amu. Only b-ions and y-ions were considered for mass calculation. Oxidation of methionine (+16 Da) was considered as a variable modification. Alkylation (+46 Da) and iTRAQ labels (+144 Da on lysines and peptide N-termini) were considered as fixed modifications. Two missed trypsin cleavages were allowed. The results of the 32 fractions were merged into a single assessment. Only peptides with Xcorr values higher than 2.0 (double charge), 2.5 (triple charge), and 3.0 (>3 charges) were retained. Monocharged peptides were not retained. In all cases, we required the peptide *P*-value to be lower than 0.001 and the ΔCn value to be above 0.1. All protein identifications were based on the detection of a minimum of two distinct peptides. With these parameters, we did not detect any false positives. Shared peptides are only counted for the protein that has over all the most matching peptides. The PepQuan algorithm embedded in Bioworks was used for reporting the intensities of 115–117 reporter ions. The mass tolerance was set to 0.35 amu.

### Peptide and protein intensity thresholds

To define quantification limits, a peptide intensity threshold value was determined. The experimental procedure has been described in Figure S1. Briefly, one PC24 sample was divided into two parts, one labeled with the 116 iTRAQ tag, and the other with the 117 tag. In theory, for every peptide, the 117/116 ratio should be equal to 1. We determined experimentally that when the intensity was above 35, the percentage of peptides with a 117/116 ratio >2 or <0.5 (i.e., false positive) was less than 5%, which was acceptable for us. Therefore, when a peptide had a normalized intensity greater than or equal to 35, it was classified as “quantifiable,” and taken into account for determining the global protein intensity. The intensity of a given protein corresponded to the sum of the intensities of all quantifiable peptides derived from this protein. Proteins were quantified when at least two peptides were detected in the iTRAQ experiment, and thus the minimal intensity value should be above 70.

### Biofilm formation assay

Biofilm formation was assayed according to a previously described protocol (Christensen et al. 1985; O'Toole et al. 1999). Precultures were performed as described above. A 5 × 10^−3^ OD_546_ bacterial suspension was prepared in 5 mL of LB, and aliquots (150 *μ*L) were then placed in the wells of a polystyrene 96-well microtiter plate (Nunc® MicroWell™ 96-well polystyrene). For each mutant and wild-type strain, three clones were tested in duplicate. After incubation for 24 h at 37°C, 200 *μ*L of PB was added into each well. Plates were then incubated at room temperature for 10 min; unattached bacterial population in PB was discarded and attached bacteria were fixed by 200 *μ*L of 100% ethanol. After an incubation of 10 min at room temperature, ethanol was removed, and the plates were incubated for 10 min at 70°C to remove residual alcohol. Thereafter, 200 *μ*L of 0.5% (w/v) crystal violet (Sigma L'Isle d'Abeau Chesnes, France) in distilled water was added into each well. After an additional incubation of 10 min, the stain was discarded, and plates were washed three times with distilled water, and then 200 *μ*L of 33% acetic acid was added into each well. The contents of eight wells (i.e., one column corresponding to one clone) were pooled in a tube containing 2 mL of 33% acetic acid. The amount of dye associated with the biofilm was measured at 590 nm using the suspension diluted 1/10.

### Statistical analysis

For biofilm-formation experiments, statistical differences were estimated by the Wilcoxon test (*n* = 6, three clones measured in duplicate). Data were considered to be statistically significant when the *P*-value was less than 0.05. Transcriptome matrix was constructed with the whole original data and analyzed by principal component analysis (PCA) (Joliffe and Morgan 1992) to reveal a statistical difference between transcriptomic data and identify main criteria involved in data variability. Statgraphic Plus 5.1 was used to perform PCA using horizontally and vertically standardized data (i.e., converted to normalized scores).

## Results

### *Pseudomonas aeruginosa* displayed a biofilm-specific gene expression when cultured on glass wool

To form *P. aeruginosa* biofilms, bacteria were cultivated in a batch system with glass wool. We first verified that these *P. aeruginosa* cultures acquired a biofilm-specific gene expression pattern distinct from the gene expression patterns of either exponential- or stationary-phase PCs, or from a combination of both. To this aim, we compared the genomic expression of *P. aeruginosa* PAO1 cells cultured under three distinct conditions: PCs in exponential growth phase (PC4), PCs in stationary growth phase (PC24), and SCs cultured for 24 h with glass wool (SC24). RNAs were prepared and transcriptome differences were monitored using the GeneChip® *P. aeruginosa* Genome Array from Affymetrix. To take into account the biological variability, three independent clones were used for each growth condition.

**Figure 1:**
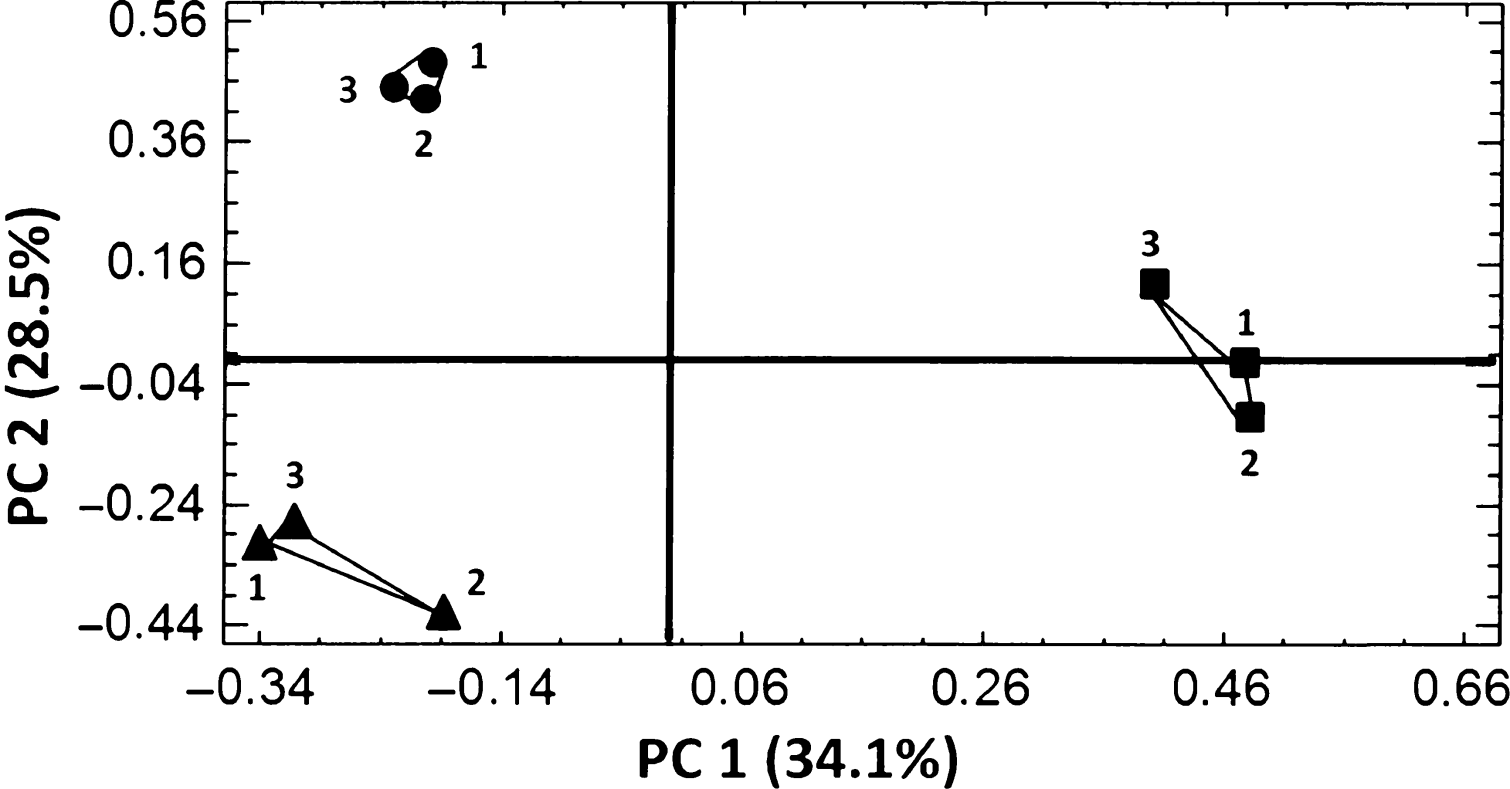
Principal component analyses (PCA) performed on normalized transcriptomic data. PCA revealed two main components: PC1 and PC2 corresponding to culture duration and growth mode, respectively. Planktonic exponentially growing PAO1 cells (PC4) are represented by squares (▪), planktonic PAO1 cells in stationary phase (PC24) are represented by circles (•), and sessile PAO1 cells (SC24) are represented by triangles (▴). The data corresponding to the three biological replicates (R1, R2, and R3) are indicated by the numbers 1, 2, and 3. Percentages indicate the importance of the PC1 and PC2 components in the variability of gene expression. The PCA results and 2D component plot were obtained using Statgraphic Plus 5.1 software.

All data obtained in the transcriptomic experiments were used to perform a statistical analysis (Fig. 1). We used PCA, a nonparametric multivariate analysis that allows graphic visualization of correlations between variables either by grouping them or separating them according to the axes of the principal components. This statistical analysis allowed highlighting similarities and differences between the three growth conditions. PCA extracted two principal components from the standardized values, each of which had eigenvalues greater than one (Kaiser 1960). These two principal components collectively accounted for 62.6% of the data variability. The first component (34.1% of the variation) differentiated the cultures in exponential phase (PC4) from the two 24-h cultures (PC24 and SC24). Therefore, this component corresponds to culture duration, and thus may reflect the proliferation status of the cells. The second component discriminated SCs from PCs in the stationary growth phase. This component accounted for 28.5% of the mRNA-level variability. These results indicate that *P. aeruginosa* SCs display a transcriptome statistically different from those of PCs. In agreement with the literature (Beloin et al. 2004), our results showed that the gene expression profile of SC24 cells resembles more that of PCs in stationary phase (PC24) than that of PCs in exponential phase (PC4). From there, we sought to define the genes specifically regulated in the biofilm state using PC24 as reference point.

### Identification of genes exhibiting a modified expression in SCs

To quantitatively assess the changes in the levels of different mRNAs in relation to the mode of growth, we used several selection criteria. We determined a threshold value and we classified genes as either overexpressed or underexpressed (see Materials and Methods section). The *P. aeruginosa* PAO1 genome is currently described as containing 5571 coding sequences (CDS) (http://www.pseudomonas.com/). Nucleotide sequences corresponding to 5548 CDS were present on the microarray and we quantified a total of 3360 (60.6%) transcripts using our threshold selection criteria. Among these 3360 genes, SCs overexpressed 92 (2.7%) and underexpressed 148 (4.4%) of them by a factor ≥2, when compared with PCs in the stationary growth phase (PC24). These 240 (7.1%) differentially expressed genes represented those likely to be involved in the biofilm phenotype.

The next step was to identify the biological pathways related to the genes whose expression was specifically modified in the SCs. To achieve this goal, we first examined the gene annotations provided by the Pseudomonas Genome Database, PseudoCAP (Winsor et al. 2009) (http://www.pseudomonas.com/). When looking at the 92 upregulated mRNAs, it appeared that genes involved in small-molecule transport (21 hits), in carbon-compound catabolism (11 hits), as well as genes coding for membrane proteins (17 hits) were well represented (Fig. S2A). In addition, functions for many of these overexpressed genes are unknown (24 hits). Regarding the genes downregulated in the SCs (148 genes), the majority are classified in the category “translation, post-translation modification, degradation” (49 hits), and most of them encoded ribosomal proteins (Fig. S2B). This result was expected because low metabolic activity and reduced growth rate are physiological traits of biofilms (Stewart and Franklin 2008). We also found genes encoding enzymes involved in the amino acids biosynthesis (12 hits), and genes belonging to the “transcription, RNA processing and degradation” category (9 hits). Again, several underexpressed genes code for proteins of unknown function (22 hits) (Fig. S2B).

Our next analysis can be considered a first step toward a comprehensive understanding of the physiology of SCs. To this aim, we scanned the Kyoto encyclopedia of genes and genomes (KEGG) database (http://www.genome.jp/kegg/). This database displays metabolic pathways that are activated or inhibited under different physiological conditions, for different organisms and species. Thus, we expected to identify metabolic pathways that were specifically different in the biofilm physiological state. Such an approach was recently reported to describe the complex metabolic systems of cells (Downs 2009). With our data, we found that overexpressed genes were mainly involved in benzoate degradation (KEGG pathway number: pae00362), the biosynthesis of siderophore group nonribosomal peptides (pae01053), and nitrogen metabolism (pae00910) (Fig. S3A). Regarding the genes downregulated in the SCs (148 genes), as previously, the majority of these mRNAs encoded ribosomal proteins (32 mRNAs classified in the category “ribosome”) (Fig. S3B). We also found genes belonging to the “purine metabolism” category (10 hits) and “pyrimidine metabolism” (6 hits) (Fig. S3B).

### Analysis of the benzoate degradation pathway

**Figure 2:**
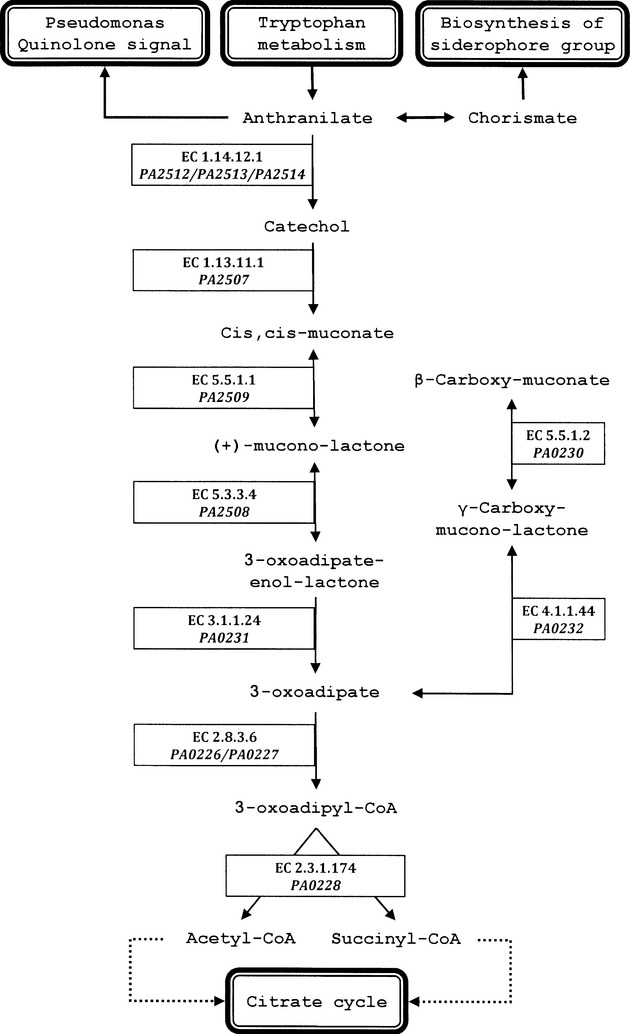
Anthranilate degradation pathway according to the KEGG database (http://www.genome.jp/kegg/).

Our KEGG database analysis revealed that several genes involved in one part of the benzoate degradation pathway (pae00362) were overexpressed in SCs (Fig. S3A). These genes are specifically involved in the seven enzymatic reactions necessary to degrade anthranilate into acetyl-CoA and succinyl-CoA, two compounds destined for the citric acid cycle (Fig. 2).

The first step of the anthranilate degradation pathway is the conversion of anthranilate into catechol. This reaction involves an oxidoreductase (EC 1.14.12.1); the products of the reaction are catechol, CO_2_, and ammonia. This oxidoreductase is made of three subunits, which are the products of the *antA*, *antB*, and *antC* (*PA2512*, *PA2513*, *PA2514*) genes belonging to the same operon. We observed increased mRNA levels for all three of these genes in the SC24 cultures (Table 1). The mRNA content of the PA2512, PA2513, and PA2514 genes was, respectively, 5.5, 20.5, and 20.8 higher in the SC24 cultures than in the PC24 cultures (Table 1).

**Table 1:** Microarray analyses of genes involved in the anthranilate degradation pathway

	Gene^1^	Name	Operon^1^	EC	Fold change	Description if available
Anthranilate degradation	PA0226	*pcaI*	47	2.8.3.6	**3.7 ± 0.7**	3-Oxoadipate CoA-transferase, subunit A
	PA0227	*pcaJ*	47	2.8.3.6	1.6 ± 0.3	3-Oxoadipate CoA-transferase, subunit B
	PA0228	*pcaF*	47	2.3.1.174	ND	β-Ketoadipyl CoA thiolase PcaF
	PA0229	*pcaT*	48		1.2 ± 0.2	Dicarboxylic acid transporter PcaT
	PA0230	*pcaB*	48	5.5.1.2	**2.7 ± 0.8**	3-Carboxy-*cis*,*cis*-muconate cycloisomerase
	PA0231	*pcaD*	48	3.1.1.24	**3.3 ± 1.4**	β-Ketoadipate enol-lactone hydrolase
	PA0232	*pcaC*	48	4.1.1.44	**4.8 ± 2.2**	γ-Carboxymuconolactone decarboxylase
	PA2507	*catA*	527	1.13.11.1	**3.3 ± 1.0**	Catechol 1,2-dioxygenase
	PA2508	*catC*	527	5.3.3.4	**2.4 ± 0.4**	Muconolactone delta-isomerase
	PA2509	*catB*	527	5.5.1.1	**2.9 ± 1.5**	Muconate cycloisomerase I
	PA2512	*antA*	528	1.14.12.1	**5.5 ± 2.6**	Anthranilate dioxygenase large subunit
	PA2513	*antB*	528	1.14.12.1	**20.5 ± 9.8**	Anthranilate dioxygenase small subunit
	PA2514	*antC*	528	1.14.12.1	**20.8 ± 9.5**	Anthranilate dioxygenase reductase
Anthranilate synthesis via the Kynurenine pathway	PA2080	*kynU*	438	3.7.1.3	1.1 ± 0.1	Kynureninase
	PA2081	*kynB*	438	3.5.1.9	0.8 ± 0.2	Kynurenine formamidase
	PA2579	*kynA*		1.13.11.11	ND	L-Tryptophan:oxygen 2,3-oxidoreductase
Anthranilate synthesis via chorismate	PA0609	*trpE*	135	4.1.3.27	0.8 ± 0.1	Anthranilate synthase component I
	PA0649	*trpG*	146	4.1.3.27	0.9 ± 0.1	Anthranilate synthase component II
	PA1001	*phnA*	215	4.1.3.27	0.9 ± 0.2	Anthranilate synthase component I
	PA1002	*phnB*	215	4.1.3.27	1.0 ± 0.2	Anthranilate synthase component II
Pseudomonas quinolone signal (PQS)	PA1003	*pqsR*			ND	LysR, transcriptional regulator
	PA0996	*pqsA*	214		0.7 ± 0.1	
	PA0997	*pqsB*	214		0.7 ± 0.1	
	PA0998	*pqsC*	214		0.4 ± 0.1	3-Oxoacyl-(acyl-carrier-protein) synthase
	PA0999	*pqsD*	214		0.5 ± 0.1	3-Oxoacyl-(acyl carrier protein) synthase III
	PA1000	*pqsE*	214		0.6 ± 0.1	

Fold change corresponds to the SC24/PC24 ratios. Results are the mean value of the nine ratios of the three biological SC24 replicates and the three biological PC24 replicates ± SD.

1 IDs of genes and operons are from the Pseudomonas Genome Database (http://www.pseudomonas.com).

ND, not determined because the intensity of the corresponding probe did not fulfill our criteria for quantification.

Bold value indicates the greater than 2.

In the second enzymatic step of this pathway, an oxidoreductase (EC 1.13.11.1) encoded by PA2507 (*catA*) converts catechol into *cis*,*cis*-muconate. Two subsequent reactions cyclize the *cis*,*cis*-muconate into (+)-mucono-lactone, which is then transformed in 3-oxoadipate-enol-lactone. These reactions are catalyzed by muconate cycloisomerase I (EC 5.5.1.1), a manganese-dependent enzyme encoded by PA2509, and by muconolactone delta-isomerase (EC 5.3.3.4) encoded by PA2508. All of these genes (PA2507, PA2508, and PA2509) belong to the same operon in *P. aeruginosa* and were upregulated in biofilms (Table 1). The corresponding mRNA levels were between 2.4- and 3.3-fold higher in SCs than in PCs.

The fifth enzymatic reaction corresponds to the hydrolysis of 3-oxoadipate-enol-lactone into 3-oxoadipate. The enzyme responsible for this reaction, EC 3.1.1.24, is encoded by PA0231 (*pcaD*) in *P. aeruginosa,* which belongs to an operon that also contains the PA0229 (*pcaT*), PA0230 (*pcaB*), and PA0232 (*pcaC*) genes. The protein PcaT is a dicarboxylic acid transporter predicted to contain 12 transmembrane helices. The PcaB and PcaC proteins catalyze two reactions that are not directly involved in anthranilate degradation. According to the benzoate degradation pathway described in the KEGG database, PcaB and PcaC degrade β-carboxy-muconate, which results in 3-oxoadipate synthesis (Fig. 2). Our transcriptomic results showed that the PA0230, PA0231, and PA0232 genes were upregulated 2.7-, 3.3-, and 4.8-fold, respectively, in SCs (Table 1).

The sixth step in anthranilate degradation transforms 3-oxoadipate into 3-oxoadipyl-CoA. The donor of CoA is succinyl-CoA, and the transferase responsible for the catalysis is a 3-oxoadipate CoA-transferase (EC 2.8.3.6). According to the KEGG database, the corresponding gene is not known for *P. aeruginosa*. However, it has been previously demonstrated that the *P. putida* enzyme is composed of two subunits encoded by the genes *pcaI* and *pcaJ* (Göbel et al. 2002). Comparison of the genomes of the *P. putida* B13 and *P. aeruginosa* PAO1 strains suggested that PA0226 and PA0227 could be the corresponding *P. aeruginosa* genes that encode the two subunits of 3-oxoadipate-CoA transferase (Göbel et al. 2002). Our study supported this hypothesis (Table 1). Transcript level for PA0226 was higher in SC24 cells than in PC24 cells (3.7-fold); PA0227 was moderately overexpressed in SC24 cells (1.6-fold). In addition, the PA0226 and PA0227 genes belong to the same operon as PA0228 (*pcaF*), which encodes the last enzyme of the pathway (EC 2.3.1.174). Altogether, these data led us to attribute the function of 3-oxoadipate CoA-transferase subunits A and B to the PA0226 and PA0227 gene products, respectively.

The final reaction is catalyzed by 3-oxoadipyl-CoA thiolase encoded by PA0228 (*pcaF*), which transforms 3-oxoadipyl-CoA into one molecule of succinyl-CoA and one molecule of acetyl-CoA. At this stage, we cannot give information about PA0228 expression because the corresponding mRNA quantity was too low to be quantified according to our criteria.

Our results raised questions about the origin of anthranilate. In Pseudomonas, this molecule is produced through the degradation of tryptophan via the kynurenine pathway or is synthesized from chorismate by two anthranilate synthases (Essar et al. 1990; Farrow and Pesci 2007). Three genes in the kynurenine pathway, PA2080 (*kynU*), PA2081 (*kynB*), and PA2579 (*kynA*) are involved in tryptophan degradation. We did not see any clear increases in the respective levels of mRNA for these genes in the SC24 samples relative to the PC24 samples (Table 1). Likewise, the four genes encoding the subunits of the two anthranilate synthases identified so far in Pseudomonas, PA0609 (*trpE*), PA0649 (*trpG*), PA1001 (*phnA*), and PA1002 (*phnB*), were not upregulated in our experimental model of biofilm formation.

Anthranilate has been shown to be a precursor for the synthesis of 2-heptyl-3-hydroxy-4-quinolone, which is referred to as the Pseudomonas quinolone signal (PQS) (Pesci et al. 1999; Cao et al. 2001). It acts as a coinducer for the transcriptional activator *pqsR* (PA1003, also known as *mvfR*), which in turn activates the *pqsABCDE* operon involved in PQS biosynthesis (Wade et al. 2005). The mRNA levels of the transcriptional regulator pqsR/mvfR were not quantified owing to their low amounts. However, the mRNAs levels of all the genes of the *pqsABCDE* operon were between 40% and 60% lower in the biofilm state. As the expression of this operon was reduced in the biofilm state, this may be an indication that the synthesis of PQS via anthranilate is not a prominent event in the SC24 cultures.

### Proteomic analysis supports the overexpression of genes involved in anthranilate degradation

To support our transcriptomic data, a complementary proteomic analysis was performed using MS and iTRAQ labels. We identified 1,571 proteins in our proteomic experiments, but only 828 (52.7%) fulfilled our quantification criteria. Among these 828 proteins, 34 (4.1%) were overexpressed by a factor ≥2 in the SCs when compared with PC24 cultures. Conversely, 4 (0.5%) proteins were downregulated by a factor ≥2.

We focused on proteins involved in anthranilate degradation pathway (Table 2). Among the thirteen proteins involved in this pathway, 10 were quantified according to our stringent criteria. Their amount in SC24 samples was increased between 1.5- and 10-fold compared with PC24 samples. Interestingly, whereas no data was obtained from transcriptomic approach, the proteomic analysis demonstrated that PA0228 product, which catalyzes the final reaction of anthranilate degradation, was more abundant in biofilm (+4.1-fold). Altogether, these proteomic data are consistent and complementary with our transcriptomic results. Combining the two approaches, our data demonstrated that all steps of anthranilate degradation were increased in 24-h-old *P. aeruginosa* SCs.

**Table 2:** Proteins of the anthranilate degradation pathway identified by the iTRAQ analyses

Protein^1^	Gene	Description	Fold change (SC24/PC24)
PA0226	*pcaI*	3-Oxoadipate CoA-transferase, subunit A	3.1
PA0227	*pcaJ*	3-Oxoadipate CoA-transferase, subunit B	4.5
PA0228	*pcaF*	β-Ketoadipyl CoA thiolase PcaF	4.1
PA0230	*pcaB*	3-Carboxy-*cis*,*cis*-muconate cycloisomerase	1.6
PA0231	*pcaD*	β-Ketoadipate enol-lactone hydrolase	1.9
PA2507	*catA*	Catechol 1,2-dioxygenase	5.4
PA2509	*catB*	Muconate cycloisomerase I	6.5
PA2512	*antA*	Anthranilate dioxygenase large subunit	10.8
PA2513	*antB*	Anthranilate dioxygenase small subunit	9.7
PA2514	*antC*	Anthranilate dioxygenase reductase	10.6

The proteins listed in this table fulfilled our quantification criteria (see Materials and Methods section). Results (SC24/PC24 ratios) are mean values of three biological replicates. Proteins exhibiting a fold change >1.5 are presented.

1 According to the Pseudomonas Genome Database (http://www.pseudomonas.com).

### Biofilm formation by mutants altered in the anthranilate degradation pathway

**Figure 3:**
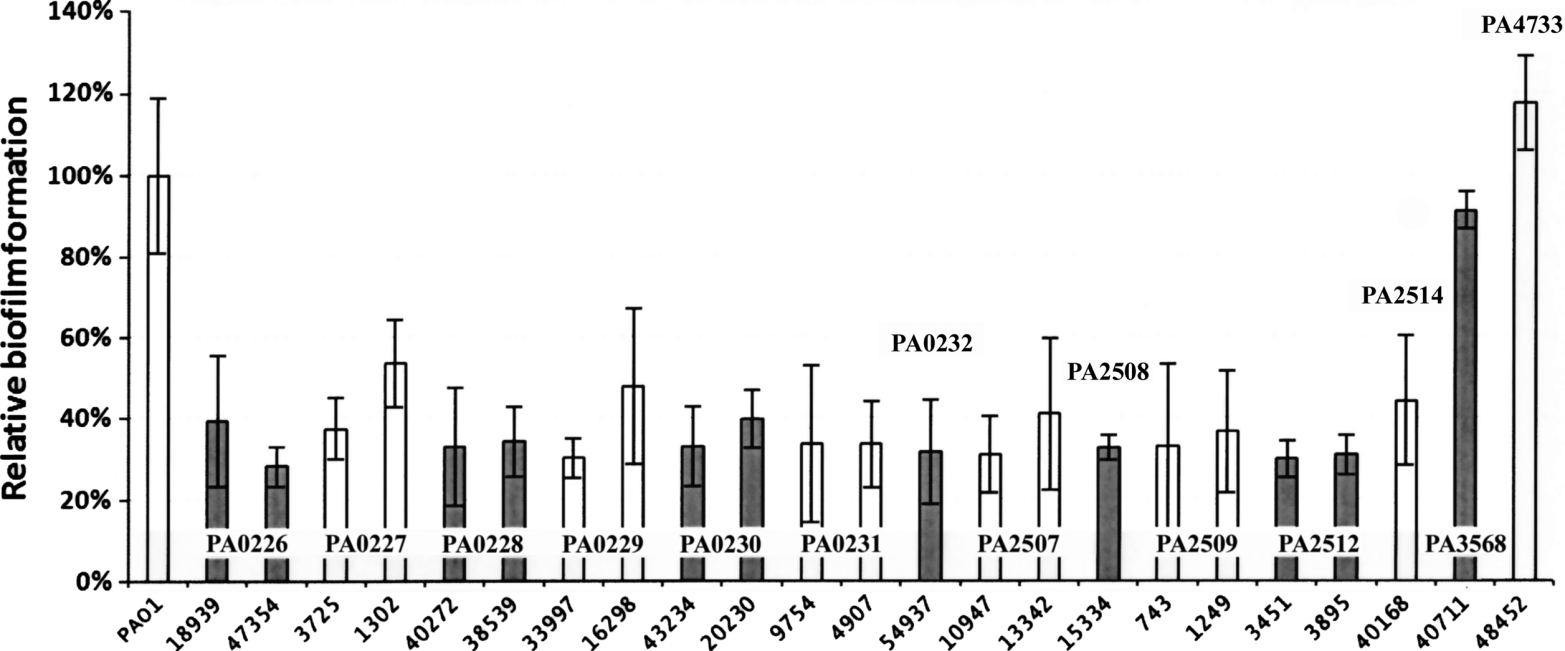
Biofilm formation by anthranilate pathway mutants. The capacity of the mutants to form biofilm was assayed by adhesion on 96-well microtiter plates. All transposon mutants and the PAO1 reference strain were provided by the University of Washington. Identity of each mutant is indicated below the histogram (http://www.gs.washington.edu/labs/manoil/libraryindex.htm) and genes are indicated by their PA numbers (http://www.pseudomonas.com). Biofilm quantities were assayed by crystal violet coloration (see Materials and Methods). According to a Wilcoxon test, all 21 mutants related to anthranilate degradation statistically produced less biofilm than PAO1 (*P* < 0.05; *n* = 6). Values are mean ± SEM of three biological replicates, each tested twice. 100% corresponds to the biofilm-formation ability of the PAO1 reference strain.

To further investigate the functional link between the anthranilate degradation pathway and biofilm formation, we tested the adhesion capacities of anthranilate degradation pathway mutants, each of which was disrupted in one of the genes that encoded an enzyme of the pathway. The transposon mutants were obtained from a *P. aeruginosa* transposon mutant library available from the University of Washington Genome Center. We studied a total of 21 mutants consisting of two distinct mutants for each of the following genes: PA0226 to PA0231, PA2507, PA2509, and PA2512; and one mutant each for the genes PA0232, PA2508, and PA2514. We were unable to find any mutants for the PA2513 gene. A prerequisite was to verify that the growth characteristics of these mutants were identical to that of the reference PAO1 strain. After 24 h of culture, growth of all mutant strains was comparable to that of reference strain (data not shown). Thereafter, biofilm formation in the 21 mutants was quantified by staining with crystal violet (Fig. 3). The mean value of the optical density measured at 590 nm for the PAO1 control strain after 24 h of culture in the polystyrene plate was 7.7, which corresponded to 100%. For the 21 transposon mutants, optical densities were between 54% and 28% of the value measured for the control strain. We checked that the diminished adhesion capacities of these mutants were not indirect effects caused by the presence of transposons. To this aim, we tested the adhesion capacities of two mutants altered for the genes *ymmS* (PA3568) and *ascB* (PA4733), each of which encodes a distinct acetyl-CoA synthetase, and which were both expressed in the sessile and PCs at similar levels. The adhesion capacities of these two mutants were similar to that of the reference strain (Fig. 3). Thus, in this in vitro system, mutations in the genes encoding the enzymes of the anthranilate degradation pathway significantly reduced the adhesion capacity of *P. aeruginosa,* which indicated that their biofilm-formation ability was impaired. All of these mutants exhibited strikingly similar diminutions in biofilm formation, showing that interruption of this metabolic pathway at any point resulted in the same biofilm-formation phenotype. The functional complementation of mutant 16298 by introducing wild-type PA0229 gene in pUCP20 vector agreed with the involvement of anthranilate degradation pathway in biofilm formation. Indeed, the complemented mutant recovered the ability to form biofilm as wild type (Fig. S4). However, anthranilate degradation pathway activity is not mandatory for biofilm formation as all mutants were still able to form biofilm to some extent.

## Discussion

*Pseudomonas aeruginosa* is an opportunistic pathogen, which infects the lungs of CF patients. Several studies indicated that in CF lungs, *P. aeruginosa* exists primarily in a biofilm rather than a planktonic state (Lam et al. 1980; Singh et al. 2000; Moreau-Marquis et al. 2008; Yang et al. 2008). Therefore, much attention has focused on understanding the mechanisms that allow these bacteria to acquire and maintain the biofilm physiology. High-throughput studies conducted so far have used different experimental models of biofilm formation: (i) after growth on a substrate made of pebbles in chemostat vessels (Whiteley et al. 2001); (ii) in a once-through continuous flow system in silicone tubing (Davies et al. 1993; Sauer et al. 2004; Hentzer et al. 2005); (iii) after growth on nitrocellulose filters placed on 20% LB agar (Waite et al. 2005, 2006); (iv) in a drip-flow plate reactor (Folsom et al. 2010); and (v) on glass wool fibers in an Erlenmeyer flask (Steyn et al. 2001; Vilain et al. 2004a). The results of these studies showed little overlap among the genes and proteins differentially expressed in the planktonic and biofilm states (An and Parsek 2007; Patell et al. 2010). Although surprising, these differences could be attributed to the experimental conditions used to culture the biofilms and/or to the physicochemical properties of the colonized surface. For example, biofilms formed by the PAO1 strain differed depending on the carbon source provided (citrate or glucose) and on the culturing system used (static or flow through) (De Kievit et al. 2001). So, we can imagine that according to its environment, *P. aeruginosa* uses different ways to make a biofilm. This hypothesis is supported by the comparative microarray analysis described by Patell and coauthors (2010). Knowledge of different metabolic pathways involved in biofilm formation is a necessary step to understand the implementation of this particular physiology.

Here, our goal was to investigate the whole-genome expression pattern of SCs cultivated on glass wool fibers, which possesses the property to bind DNA. The eDNA has been described as essential for establishing the three-dimensional architecture of biofilm (Whitchurch et al. 2002), and as a relevant component of antibiotic resistance (Mulcahy et al. 2008). The glass wool substrate offered other advantages. It is simple to use and the surface area offered for biofilm development is large enough to obtain sufficient biomass for proteomic analyses (Steyn et al. 2001).

Using this experimental system, we performed a transcriptomic study. PCA analyses of microarray data showed that SCs cultured for 24 h on glass wool have a specific pattern of genome expression. A major feature revealed by the transcriptomic analysis of SCs was the overexpression of genes controlling anthranilate degradation. Anthranilate is an important intermediate in several metabolic pathways in *P. aeruginosa*. It can be synthesized via two pathways: through the degradation of tryptophan via the kynurenine pathway or synthesized from chorismate by two anthranilate synthases (Essar et al. 1990; Farrow and Pesci 2007). Once formed, anthranilate can be converted by several enzymes into succinate, and thus can be used for energy metabolism, or it can be used for PQS synthesis (Farrow and Pesci 2007). Several studies have been devoted to understanding the interplay between these different pathways. It has been recently shown that the *ant* and *cat* operons were induced by acyl-homoserine lactone (acyl-HSL) in *P. aeruginosa* PAO1, and that this induction occurred in the absence of the *rhl* and *las* quorum sensing systems (Chugani and Greenberg 2010). The activation of the *ant* and *cat* operons by acyl-HSL proceeds through AntR (PA2511) (Oglesby et al. 2008), which requires anthranilate as a coactivator. Induction of anthranilate degradation coincided with an increase in the PQS biosynthesis pathway, which consumed anthranilate (Chugani and Greenberg 2010). This study demonstrated that the same pool of intracellular anthranilate can be simultaneously used for energy metabolism and synthesis of the PQS molecule, and that this pool was replenished by the degradation of tryptophan via the kynurenine pathway. A link between anthranilate and PQS production was also observed when planktonic cultures of the *P. aeruginosa* PA14 strain were grown in a medium supplemented with CF sputum (Palmer et al. 2005). In this case, induction of the *phnAB* operon, which is involved in the synthesis of anthranilate from chorismate, coincided with the induction of the *pqsA-E* operon, and suggested that the anthranilate produced was used to synthesize the PQS molecule. In addition, it has been shown that the repression of anthranilate degradation by the regulatory PrrF RNAs is important for allowing PQS biosynthesis in iron-limiting environments (Oglesby et al. 2008). Altogether, these studies demonstrate a central role for anthranilate in the production of PQS in *P. aeruginosa*.

Our study revealed a new physiological response concerning anthranilate utilization during biofilm formation. Indeed, our results suggest that anthranilate is used for energy metabolism rather than PQS production. In our model of biofilm formation, we observed strong inductions, in terms of elevated mRNA and protein levels, for several genes belonging to the *catABC*, *antABC*, *pcaIJF*, and *pcaTBDC* operons, which encode enzymes of the anthranilate degradation pathway. Conversely, the genes of the *pqs* operon were downregulated, suggesting that the anthranilate pool was mainly used for energy production through the tricarboxylic acid cycle. Finally, none of the genes encoding the enzymes involved in anthranilate biosynthesis were upregulated in the glass wool biofilms, suggesting that anthranilate synthesis was occurring at a steady rate. A recent study investigating the gene expression profiles of *P. aeruginosa* biofilm-like structures at air–liquid interfaces, revealed strong inductions of the *cat* and *ant* operons that were not associated with induction of the PQS system (Yamamoto et al. 2011). Taken together, these data also suggest that the anthranilate pool is used as a source of energy during biofilm formation. In our model, because we did not observe any upregulation of genes involved in the anthranilate biosynthesis, we can postulate that the intracellular anthranilate pool will be rapidly depleted. Thus, anthranilate could be a transient source of energy in SCs, which may explain why the induction of this pathway has not been reported for other models of biofilm formation. Alternatively, the induction of this pathway might depend on the carbon source present in the growth medium.

Anthranilate is a metabolic branch point and its use in *P. aeruginosa* may fluctuate depending on growth conditions (Choi et al. 2011). It was reported that the induction and repression of genes involved in anthranilate metabolism are tuned along with growth by the antagonistic interplay of quorum sensing regulators. Here, we revealed for the first time, a new feature of anthranilate metabolism in connection with the formation of *P. aeruginosa* biofilm. Indeed SCs grown on glass wool exhibited a particular response with respect to anthranilate degradation. In addition, using a different model where biofilm formation took place in 96-well polystyrene plates (Christensen et al. 1985; O'Toole et al. 1999), we showed that mutant strains harboring different inactive genes involved in this pathway developed less biofilm than the reference PAO1 strain. This result confirmed that in our experimental conditions, an intact anthranilate degradation pathway was necessary to establish or maintain a biofilm state in *P. aeruginosa*.

## Supporting Information

**Figure S1.** Determination of the peptide intensity threshold to be considered in the quantitative analysis. The peptide intensity threshold value was determined by dividing one PC24 sample into two parts, one labeled with the 116 iTRAQ tag and the other with the 117 tag. In theory, for every peptide, the 117/116 ratio should be equal to 1. From the 116 and 117 normalized intensities, we built a new matrix where each of the 2079 peptides identified in MS was characterized by its 116 intensity and the corresponding 117/116 ratio. Peptides were ranked in the ascending order relative to their intensity for 116, and then grouped in class scope fixed to five units of normalized intensity. Each class was characterized by the 116 populations (*n*), the average of 116 intensities and the percentage of ratio above or under a factor of two within the class. These last two characteristics were used to draw the graph above, which showed that when the peptide intensity was above 35, the percentage of peptides with a 117/116 ratio >2 or <0.5 (i.e., false positive) was less than 5%. Therefore, when a peptide had a normalized intensity greater than or equal to 35, it was classified as “quantifiable” and taken into account for determining the global protein intensity. Experimental data are represented by the dotted line, and the fitted curve is indicated by the solid line.

**Figure S2.** Schematic representation of modified “functions” in 24-h-old sessile PAO1 cells (SC24) according to transcriptomic analysis. Functions were identified according to the Pseudomonas Genome Database (http://www.pseudomonas.com). The numerical scale indicates the numbers of mRNA overexpressed (A, red lines) or underexpressed (B, green lines) in SC24 compared to PC24 planktonic PAO1 cells.

**Figure S3.** Schematic representation of modified “pathways” from 24-h-old sessile PAO1 cells (SC24) according to transcriptomic analysis. Pathways were identified according to KEGG Database (http://www.genome.jp/kegg/). The numerical scale indicates the numbers of mRNA overexpressed (A, red lines) or underexpressed (B, green lines) in SC24 compared with PC24 planktonic PAO1 cells.

**Figure S4.** Functional complementation of the mutant 16298 (ΔPA0229) restores its capacity to form biofilm. The PA0229 coding sequence with its own promoter was amplified from PAO1 genomic DNA and inserted in the multicloning site of the pUCP20 vector (kindly provided by Pr. Schweizer) via a double digestion *EcoR*I-*BamH*I. pUCP20 is a shuttle vector *Escherichia coli* – *Pseudomonas aeruginosa*. The recombinant vector was cloned in *E. coli* on LB plates with ampicillin (100 *µ*g/mL). The molecular construction was verified by sequencing. Then the pUCP20 expressing PA0229 as well as the empty vector were introduced into the *P. aeruginosa* mutant 16298 (insertion of transposon in gene PA0229 = ΔPA0229) by chemical transformation. Carbenicillin-resistant (200 *µ*g/mL) colonies were selected and tested for biofilm assay. The capacity of PAO1, mutant 16298 with or without pUCP20 and of the complemented mutant 16298 (containing pUCP20 expressing PA0229) to form biofilm was assayed by adhesion on 96-well microtiter plates. Biofilm quantities were determined by crystal violet staining. According to a Wilcoxon test (*P* < 0.05; *n* = 6; ***** indicates a statistical difference), ΔPA0229 and ΔPA0229 + pUCP20 significantly produced less biofilm than PAO1 whereas biofilm quantities formed by ΔPA0229 complemented were not statistically different from PAO1 ones. Values are mean ± SEM of three biological replicates, each tested twice. 100% corresponds to the biofilm formation ability of the PAO1 reference strain.
