# Supplementary material for: Evidence for the involvement of the anthranilate degradation pathway in Pseudomonas aeruginosa biofilm formation
Source: Microbiologyopen. 2012 Sep 1;1(3):326–39. doi: 10.1002/mbo3.33 (PMC3496976; doi:10.1002/mbo3.33)
Supplement: Supplementary file 1 [file mbo30001-0326-SD1.doc]

**Appendix / Supporting information**


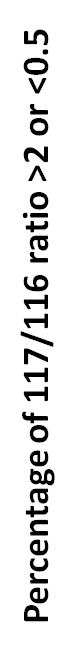


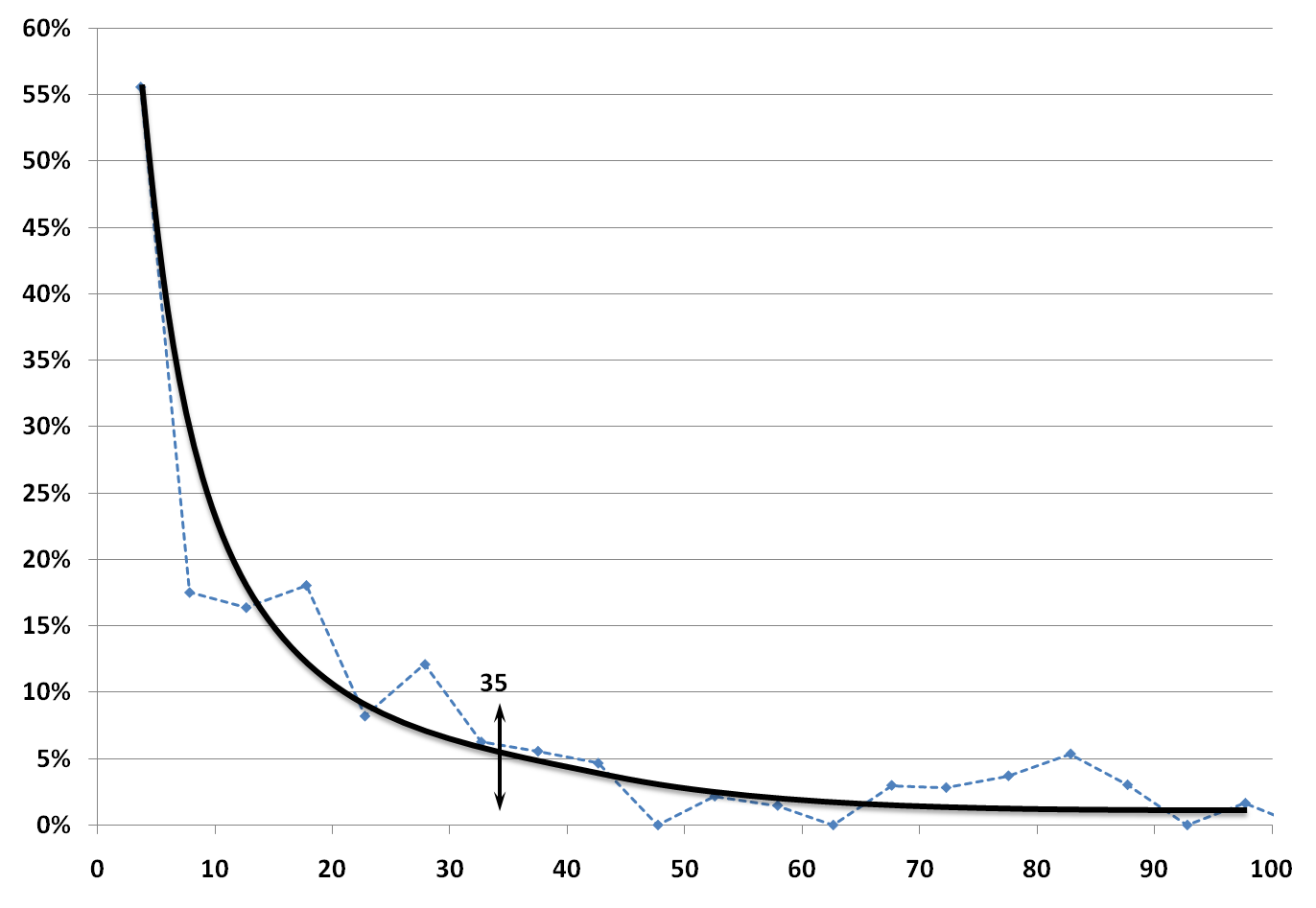

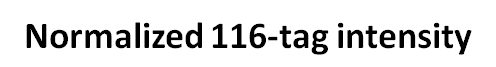


**Fig. S1:** **Determination of the peptide intensity threshold to be considered in the quantitative analysis**. The peptide intensity threshold value was determined by dividing one PC24 sample into two parts, one labeled with the 116 iTRAQ tag and the other with the 117 tag. In theory, for every peptide, the 117/116 ratio should be equal to 1. From the 116 and 117 normalized intensities, we built a new matrix where each of the 2,079 peptides identified in MS was characterized by its 116 intensity and the corresponding 117/116 ratio. Peptides were ranked in the ascending order relative to their intensity for 116, and then grouped in class scope fixed to five units of normalized intensity. Each class was characterized by the 116 populations (n), the average of 116 intensities and the percentage of ratio above or under a factor of two within the class. These last two characteristics were used to draw the graph above, which showed that when the peptide intensity was above 35, the percentage of peptides with a 117/116 ratio >2 or < 0.5 (i.e. false positive) was less than 5%. Therefore, when a peptide had a normalized intensity greater than or equal to 35, it was classified as "quantifiable" and taken into account for determining the global protein intensity. Experimental data are represented by the dotted line, and the fitted curve is indicated by the solid line.
