# Supplementary material for: Evidence for the involvement of the anthranilate degradation pathway in Pseudomonas aeruginosa biofilm formation
Source: Microbiologyopen. 2012 Sep 1;1(3):326–39. doi: 10.1002/mbo3.33 (PMC3496976; doi:10.1002/mbo3.33)
Supplement: Supplementary file 3 [file mbo30001-0326-SD3.doc]

**Appendix / Supporting information**


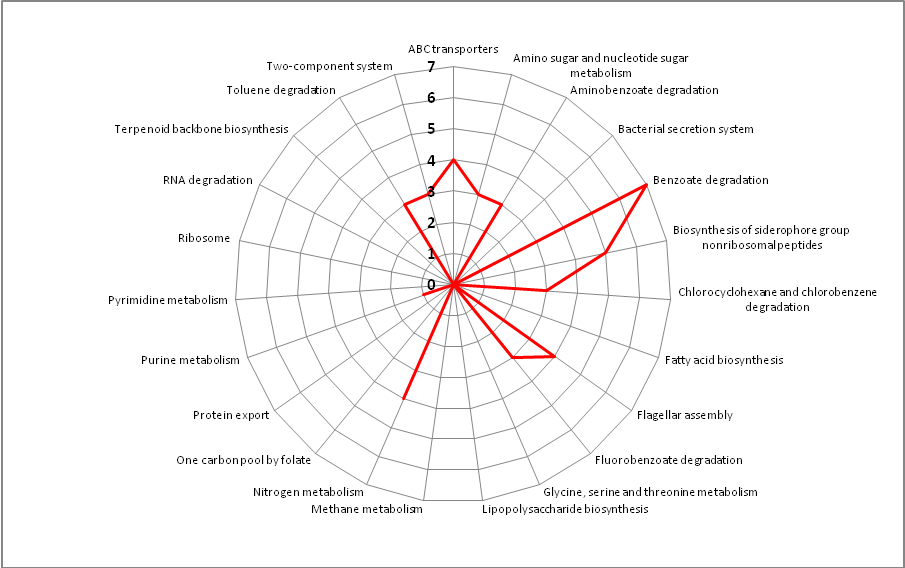


**A**


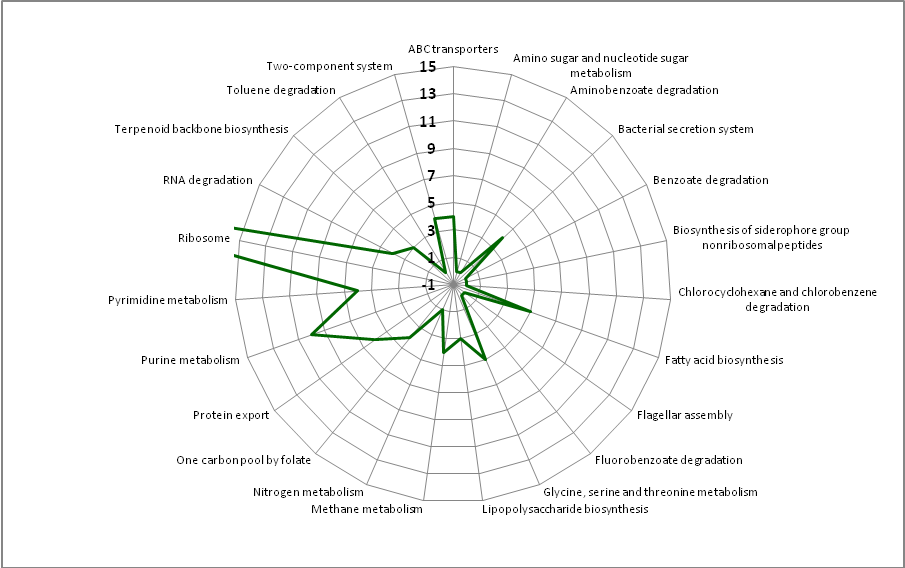


**32**

**B**

**Fig. S3**: **Schematic representation of modified “pathways” from 24h-old sessile PAO1 cells (SC24) according to transcriptomic analysis**. Pathways were identified according to KEGG Database ([www.genome.jp/kegg/](http://www.genome.jp/kegg/)). The numerical scale indicates the numbers of mRNA overexpressed (**A**, red lines) or underexpressed (**B**, green lines) in SC24 compared to PC24 planktonic PAO1 cells.
