# Supplementary material for: Evidence for the involvement of the anthranilate degradation pathway in Pseudomonas aeruginosa biofilm formation
Source: Microbiologyopen. 2012 Sep 1;1(3):326–39. doi: 10.1002/mbo3.33 (PMC3496976; doi:10.1002/mbo3.33)
Supplement: Supplementary file 4 [file mbo30001-0326-SD4.doc]

**Appendix / Supporting information**


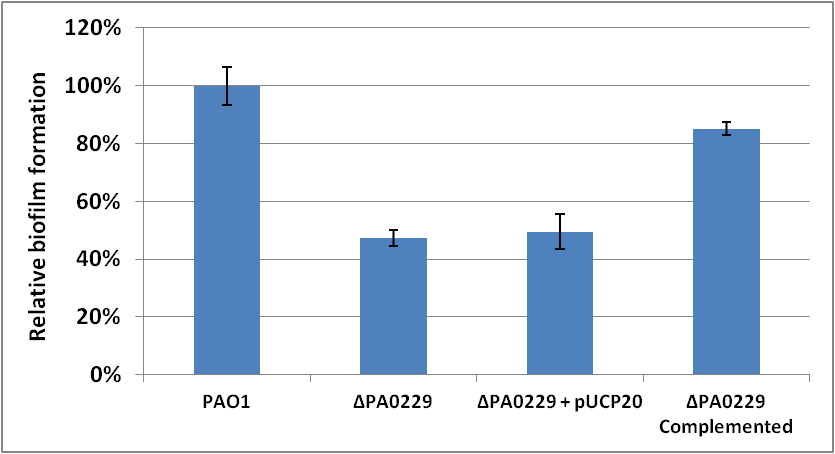


*

*

**Fig. S4:** **Functional complementation of the mutant 16298 (ΔPA0229) restores its capacity to form biofilm.**

The PA0229 coding sequence with its own promoter was amplified from PAO1 genomic DNA and inserted in the multicloning site of the pUCP20 vector (kindly provided by Pr. Schweizer) via a double digestion *EcoR*I-*BamH*I. pUCP20 is a shuttle vector *E. coli* – *P. aeruginosa*. The recombinant vector was cloned in *E. coli* on LB plates with ampicillin (100 µg/mL). The molecular construction was verified by sequencing. Then the pUCP20 expressing PA0229 as well as the empty vector were introduced into the *P. aeruginosa* mutant 16298 (insertion of transposon in gene PA0229 = ΔPA0229) by chemical transformation. Carbenicillin-resistant (200 µg/mL) colonies were selected and tested for biofilm assay.

The capacity of PAO1, mutant 16298 with or without pUCP20 and of the complemented mutant 16298 (containing pUCP20 expressing PA0229) to form biofilm was assayed by adhesion on 96-well microtiter plates. Biofilm quantities were determined by crystal violet staining. According to a Wilcoxon test (p<0.05; n=6; ***** indicates a statistical difference), ΔPA0229 and ΔPA0229+pUCP20 significantly produced less biofilm than PAO1 whereas biofilm quantities formed by ΔPA0229 complemented were not statistically different from PAO1 ones. Values are mean ± SEM of three biological replicates, each tested twice. 100% corresponds to the biofilm formation ability of the PAO1 reference strain.
